# Myocardial Structural and Biological Anomalies Induced by High Fat Diet in *Psammomys obesus* Gerbils

**DOI:** 10.1371/journal.pone.0148117

**Published:** 2016-02-03

**Authors:** Abdelhamid Sahraoui, Céline Dewachter, Geoffrey de Medina, Robert Naeije, Souhila Aouichat Bouguerra, Laurence Dewachter

**Affiliations:** 1 Laboratory of Physiology and Pharmacology, Faculty of Medicine, Université Libre de Bruxelles, Brussels, Belgium; 2 Team of Cellular and Molecular Physiopathology, Faculty of Biological Sciences, Houari Boumediene University of Sciences and Technology, El Alia, Algiers, Algeria; Temple University, UNITED STATES

## Abstract

**Background:**

*Psammomys obesus* gerbils are particularly prone to develop diabetes and obesity after brief period of abundant food intake. A hypercaloric high fat diet has been shown to affect cardiac function. Here, we sought to determine whether a short period of high fat feeding might alter myocardial structure and expression of calcium handling proteins in this particular strain of gerbils.

**Methods:**

Twenty *Psammomys obesus* gerbils were randomly assigned to receive a normal plant diet (controls) or a high fat diet. At baseline and 16-week later, body weight, plasma biochemical parameters (including lipid and carbohydrate levels) were evaluated. Myocardial samples were collected for pathobiological evaluation.

**Results:**

Sixteen-week high fat dieting resulted in body weight gain and hyperlipidemia, while levels of carbohydrates remained unchanged. At myocardial level, high fat diet induced structural disorganization, including cardiomyocyte hypertrophy, lipid accumulation, interstitial and perivascular fibrosis and increased number of infiltrating neutrophils. Myocardial expressions of pro-apoptotic Bax-to-Bcl-2 ratio, pro-inflammatory cytokines [interleukin (IL)-1β and tumor necrosis factor (TNF)-α], intercellular (ICAM1) and vascular adhesion molecules (VCAM1) increased, while gene encoding cardiac muscle protein, the alpha myosin heavy polypeptide (MYH6), was downregulated. Myocardial expressions of sarco(endo)plasmic calcium-ATPase (SERCA2) and voltage-dependent calcium channel (Cacna1c) decreased, while protein kinase A (PKA) and calcium-calmodulin-dependent protein kinase (CaMK2D) expressions increased. Myocardial expressions of ryanodine receptor, phospholamban and sodium/calcium exchanger (Slc8a1) did not change.

**Conclusions:**

We conclude that a relative short period of high fat diet in *Psammomys obesus* results in severe alterations of cardiac structure, activation of inflammatory and apoptotic processes, and altered expression of calcium-cycling determinants.

## Introduction

In its native environment, *Psmammomys obesus* gerbil is healthy, with a metabolic-endocrine system that is adjusted to desert life with a low caloric diet (composed of halophilic plant staple). However, on a high energetic diet, *Psammomys obesus* is predisposed to develop rapidly diabetes and obesity [1], mainly due to the feedback inhibition of the insulin signaling pathway, responsible for the alteration of glucose transport but not lipogenesis in the tissue [2]. This pathologic adaptation to nutrient excess represents a reliable experimental model for studying the mechanisms underlying the predisposition to develop insulin resistance and metabolic syndrome in humans who evolve from scarcity to abundant food intake [2].

Under physiological conditions, the heart relies on β-oxidation of long chain fatty acids to generate ≅ 60–70% of the adenosine triphosphate (ATP) needed for myocardial function [3]. Since cardiomyocytes have little capacity for *de novo* synthesis and storage of fatty acids [4], substrate uptake must be efficient to rapidly match energy demands. However, over-abundance of fatty acids from a high fat diet may result in excessive myocardial uptake, storage and oxidation of free fatty acids [5], which has been associated with obesity-related cardiomyopathy and increased risks for heart failure [6, 7]. Indeed, despite increased fatty acid oxidation, the excessive availability of fatty acids may lead to an imbalance between their uptake and their oxidation, resulting in an increased deposition of potentially toxic lipids in the heart [8, 9]. Myocardial lipid accumulation has been implicated in the deterioration of cardiac function and efficiency [10–12] and in the development of cardiomyopathy through different ways, including lipid-induced apoptosis [13] and fibrosis [14, 15]. Upregulation of fatty acid oxidation may also result in disturbed myocardial metabolism and increased production of reactive oxygen species (ROS) [16]. This may lead to increased stress for the sarco(endo)plasmic reticulum, resulting in disturbed calcium (Ca^2+^) handling, impaired cardiac muscle contraction/relaxation coupling and heart failure [17]. While much attention has been paid to the clinical effects of high fat diets on the incidence and the mechanisms underlying coronary heart disease and atherosclerosis, little is known on their effects on the development and the progression of heart failure.

In the present study, we, therefore, investigated the effects of 16-week administration of a hypercaloric high fat diet in *Psammomys obesus* gerbils. The results show that this relatively short period of high fat dieting is associated with marked changes in myocardial structure and biological alterations indicating activation of inflammation and apoptosis, and altered expression of proteins implicated in myocardial Ca^2+^ handling, with decreased expression of SERCA2 and increased expressions of PKA and CaMK2D. There were no changes in myocardial expressions of ryanodine receptor, phospholamban and Slc8a1.

## Methods

### Animals

The present study was approved by the Institutional Animal Care and Use Committee of the University of Bab Ezzouar (Algiers, Algeria; Permit number for the present research project: F00220110048) and has been achieved according to the Executive Decree n°10–90 completing the Executive Decree n°04–82 of the Algerian Government, establishing the terms and modalities of animal welfare in animal facilities.

Briefly, *Psammomys obesus* gerbils were trapped in the semi-desert Algerian region of Beni-Abbes (30°7 latitude north and 2°10 longitude west) at dawn in order to place the trap before the first release of the animal. The trap is a metal cage with a door that is latched at the slightest movement. The authorization to capture the animals in desert region was given by the Ministry of Higher Education (Algeria).

### Animal model

After acclimation to laboratory conditions in animal facilities (25°C, 50%-relative air humidity and 12:12 hours light and dark cycles) during two weeks, the animals were fed with their natural food composed of halophilic plants [18, 19]. Thereafter, sex-matched eight-week old gerbils (weighting 94 ± 6 g and with a male-to-female ratio: 1/3) were randomized in two groups regarding the type of food they received: control animals were fed with normal diet of 50 g natural halophilic plants (*Salicornia*; composition of the halophilic plants: water 80.8 g; mineral salts 6.9 g; lipids 0.4 g; proteins 3 g; carbohydrates 8.4 g; and 45–50 kcal/100 g) and the other group received a high fat diet, comprising the halophilic plants (50 g) plus the daily addition of one-quarter (5 g) of cooked egg yolk (composition of the cooked egg yolk: water 40–46 g; proteins 13.5–17.5 g; carbohydrates 0.2 g; lipids 30–31 g; cholesterol 1.2–1.3 g; and 370–400 kcal/100 g). After sixteen weeks of diet administration, the animals were killed by decapitation. Myocardial tissue was immediately sampled, snap-frozen in liquid nitrogen and stored at –80°C for biological analyses (n = 10 in each group) or after three days of fixation in Bouin’s aqueous solution, embedded *in toto* in paraffin for histopathological evaluation (n = 10 in each group).

### Biochemical analysis

The animals were bled from the retro-orbital venous plexus at baseline time and after sixteen-week feeding. Blood samples were immediately centrifuged at 3000 rpm on dried tubes. Glucose, triglyceride and total cholesterol concentrations were determined with BIOSYSTEM kits (Barcelona, Spain) in plasma samples, according to manufacturer’s instructions. Sera were used for the assay of lipoproteins on agarose gel by the method of Kalwakami [20].

### Cardiac morphometry

Five-micrometer myocardial sections were taken along the longitudinal axis of the heart and stained with hematoxyllin-eosin for overall morphological analysis, as previously described [21]. Mean cross-sectional areas of cardiomyocytes were calculated by measuring at least 50 cells for each myocardial sample (light microscopy, x400 magnification). Two individuals independently assessed morphological differences in a blind fashion. Immunohistochemical analysis was performed using Image J analysis software.

Masson’s Trichrome staining was used to assess the presence of collagen accumulation and fibrosis within the myocardial sections.

### Immunohistochemistry—Evaluation of inflammatory cell infiltration

Five-micrometer sections were dewaxed and progressively rehydrated. For antigen retrieval, sections were incubated in target retrieval buffer (Dako, Glostrup, Denmark) and heated in a 95°C bath for 20 minutes. Endogenous peroxidase activity was quenched with hydrogen peroxide in phosphate buffered saline (PBS) (5%) for 10 minutes, and the sections were blocked by incubation with bovine serum albumin in PBS (3%) for 30 minutes. Sections were allowed to react overnight at 4°C with monoclonal mouse anti-human myeloperoxidase antibody (MPO; 1:100 diluted in PBS; R&D Systems, Minneapolis, MN). Sections were then incubated with biotinylated anti-rabbit IgG (Dako, Le Perray en Yvelines, France) and subsequently with streptavidin–peroxidase (Dako). Antibody binding was detected with a liquid DAB (diaminobenzidine) substrate kit (AbCys Biology, Paris, France). The appearance of a brown reaction product was observed under a light microscope. Nuclei were counterstained with hematoxylin (Sigma-Aldrich, St Louis, MO) and mounted. Negative controls run without the primary antibody were tested and were found to be negative.

In each myocardial tissue slide, ten microscopic intra-myocardial fields (light microscopy, x400 magnification) were randomly chosen. In these fields, the numbers of extravascular neutrophils (MPO-positive) were counted. Intravascular inflammatory cells were excluded. Total surface of each sample was then measured using Image J software. The average numbers of intramyocardial extravascular inflammatory cells per mm^2^ were then calculated as the total score for each specimen according to Begieneman et al. [22]. Two investigators, who were blinded to the assigned group, did MPO-positive cell counts.

### Real–time quantitative polymerase chain reaction (RTQ-PCR)

Total RNA was extracted from snap-frozen myocardial tissue using the QIAGEN RNeasy® Mini kit (QIAGEN, Hilden, Germany), according to the manufacturer’s instructions. RNA concentration was determined by standard spectrophotometric technique and RNA integrity was assessed by visual inspection of GelRed (Biotium, Hayward, California)-stained agarose gels. Reverse transcription was performed using random hexamer primers and Superscript II Reverse Transcriptase (Invitrogen, Merelbeke, Belgium), according to the manufacturer’s instructions. For RTQ-PCR, sense and antisense primers were designed using Primer3 program for *rattus norvegicus* pro-alpha1 chains of collagen type 1 (Col1A1) and 3 (Col3A1), Bcl2-associated X protein (Bax), B-cell lymphoma 2 (Bcl-2), interleukin-1beta (IL-1β), interleukin-6 (IL-6), tumor necrosis factor-alpha (TNF-α), intercellular adhesion molecule 1 (ICAM1), vascular cell adhesion molecule 1 (VCAM1), hypoxia-inducible factor-1alpha (HIF-1α), sarco(endo)plasmic reticulum Ca^2+^ ATPase 2 (SERCA2), ryanodine receptor 2 (RYR2), voltage-dependent L-type Ca^2+^ channel alpha1c subunit (Cacna1c), solute carrier family 8 (sodium/calcium exchanger) member 1 (Slc8A1), myosin heavy chain isoform 6 (MYH6 or α-MHC), Ca^2+^/calmodulin-dependent protein kinase II delta (CaMK2D) and hypoxanthine phosphoribosyltransferase 1 (HPRT1) mRNA sequences (Table 1). To avoid inappropriate amplification of residual genomic DNA, intron-spanning primers were selected when exon sequences were known. For each sample, amplification reaction was performed in triplicate using SYBRGreen PCR Master Mix (Quanta Biosciences, Gaithersburg, MD, USA), specific primers and diluted template cDNA. Result analysis was performed using a Cycler System (BioRad Laboratories). Relative quantification was achieved with the comparative 2-ΔΔCt method by normalization with the housekeeping gene (HPRT1). Results were expressed as relative fold increase over the mean value of relative mRNA expression of 16-week normal diet control group arbitrary fixed to 1.

**Table 1:** Primers used for real-time quantitative polymerase chain reaction in myocardial tissue from *Psammomys obesus* gerbils.

Genes		Primer Sequences
**Pro-alpha1 collagen type 1 (Col1A1)**	*Sense*	5'- GGTTTCAGTGGTTTGGATGG -3'
	*Antisense*	5'- AGCTCCATTTTCACCAGGAC -3'
**Pro-alpha1 collagen type 3 (Col3A1)**	*Sense*	5'-AGGACAAAGAGGGGAACCTG -3'
	*Antisense*	5'- CCACCAGGACTGCCATTATT -3'
**Bax**	*Sense*	5'- CGTGGTTGCCCTCTTCTACT -3'
	*Antisense*	**5'- TCACGGAGGAAGTCCAGTGT -3'**
**Bcl-2**	*Sense*	5'- GTGGACAACATCGCTCTGTG -3'
	*Antisense*	**5'- CATGCTGGGGCCATATAGTT -3'**
**Interleukin-1beta (IL-1β)**	*Sense*	5'- AAAAATGCCTCGTGCTGTCT -3'
	*Antisense*	**5'- TCGTTGCTTGTCTCTCCTTG -3'**
**Tumor necrosis factor-alpha (TNF-α)**	*Sense*	5'-ATGGGCTCCCTCTCATCAGT-3'
	*Antisense*	5'-GCTTGGTGGTTTGCTACGAC-3'
**Interleukin-6 (IL-6)**	*Sense*	5'- CCACCAGGAACGAAAGTCA -3'
	*Antisense*	**5'- TCAGTCCCAAGAAGGCAACT -3'**
**Intercellular adhesion molecule (ICAM)1**	*Sense*	5’- ACGACGCTTCTTTTGCTCTG-3’
	*Antisense*	5’- GCAGTCCTTCTTGTCCAGGT-3’
**Vascular cell adhesion molecule (VCAM)1**	*Sense*	5’- GCCCAAAATGAAGATGAACC -3’
	*Antisense*	5’- GACGCTCCCAAAAGAAAAGA -3’
**Hypoxia-inducible factor-alpha (HIF-1α)**	*Sense*	5'- CAGAGGAAGCGAAAAATGGA-3'
	*Antisense*	5'-ACATAGTAGGGGCACGGTCA-3'
**Sarco(endo)plasmic reticulum calcium ATPase 2 (SERCA2)**	*Sense*	5'- GCAGGTCAAGAAGCTCAAGG -3'
	*Antisense*	5'- CTCGATCACAAGTTCCAGCA -3'
**Ryanodine Receptor 2 (RYR2)**	*Sense*	5'- GGAACTGACGGAGGAAAGTG -3'
	*Antisense*	5'- GAGACCAGCATTTGGGTTGT -3'
**Voltage-dependent L-type Ca**^**2+**^ **channel alpha1c subunit (Cacna1c)**	*Sense*	5'- CCTATTTCCGTGACCTGTGG -3'
	*Antisense*	5'- GGAGGGACTTGATGGTGTTG -3'
**Solute carrier family 8 (Na**^**+**^**/Ca**^**2+**^ **exchanger) member 1 (Slc8A1)**	*Sense*	5'- GAGATTGGAGAACCCCGTCT -3'
	*Antisense*	5'- AGTGGCTGCTTGTCATCGTA -3'
**Myosin heavy chain isoform 6 (MYH6 or α-MHC)**	*Sense*	5’-TCTCTTCCAGCCCTCTTTCA -3’
	*Antisense*	5’- TGTTGGCGTACAGGTCTTTG-3’
**Calcium/calmodulin-dependent protein kinase 2 delta** (CaMK2D)	*Sense*	5’- ATCCACAACCCTGATGGAAA -3’
	*Antisense*	5’- GCTTTCGTGCTTTCACGTCT -3’
**Hypoxanthine phosphoribosyltransferase 1 (HPRT1)**	*Sense*	5’- ACAGGCCAGACTTTGTTGGA -3’
	*Antisense*	5’- TCCACTTTCGCTGATGACCAC -3’

### Western blotting

Proteins were extracted from snap-frozen myocardial tissue by homogenization in an appropriate ice-cold lysis buffer [Tris-HCl pH 7.4, NaCl, NaF, sodium pyrophosphate (all at 25 mM), sodium vanadate (1 mM), EDTA, EGTA (both at 2.5 mM), phenylmethylsulfonyl fluoride (1 mM), aprotinine, leupeptine (both at 5 μg.mL^−1^), SDS, deoxycholate and NP-40 (all at 0.50%)], as previously described [23]. After centrifugation, protein concentration was determined using the method of Bradford [24]. Protein extracts (50 μg) were resolved on 4–12% NuPage Bis-Tris gels (Invitrogen, Carlsbad, CA, USA) and electro-transferred to nitrocellulose membranes (Invitrogen, Carlsbad, CA, USA). After blocking with 5% non-fat milk in TBS [Tris–HCl (pH 8.0); NaCl 150 mM] for 1 hour at room temperature, the membranes were incubated with rabbit monoclonal anti-SERCA2, rabbit polyclonal anti-phospholamban and rabbit monoclonal anti-catalytic subunit isoform (c-α) of protein kinase A (PKA) (all diluted at 1:1000; Cell Signaling Biotechnology Inc., Danvers, MA, USA) antibodies at 4°C overnight with rocking. After incubation with secondary HRP-conjugated anti-rabbit antibody (1:50 000; Millipore, Temecula, CA, USA), immunoreactive bands were detected using SuperSignal® WestPico Chemiluminescent substrate (ThermoScientific, Rockford, IL, USA) and quantified with a computed optical method using the Bio 1D software (Vilber Lourmat, France). Relative quantification was performed by normalization with β-actin (Sigma-Aldrich, St Louis, MO, USA).

### Statistical analysis

All data were expressed as mean ± standard error of the mean (SEM). Statistical analyses were performed using StatView 5.0 Software. Differences between groups were determined by performing one-way analysis of variance (one-way ANOVA). When the F ratio of this analysis resulted in a p value of less than 0.05 critical value, comparisons were made with a modified two-tailed Student t-test. A p value of p<0.05 was considered statistically significant.

## Results

### General features of experimented animals–Body weight and biochemical analysis

At baseline, the body weight and the biochemical parameters were similar between the two study groups of *Psammomys obesus* gerbils (Table 2). Sixteen-week high fat diet increased the body weight (by 28% compared to baseline and by 6% compared to normal diet) and the plasma levels of lipids, including triglycerides, total cholesterol (TC), low-density (LDL-c) and high-density lipoprotein cholesterol (HDL-c) (Table 2). The ratio of LDL-c to HDL-c was 4-fold increased after 16-week high fat compared to normal diet (LDL-c/HDL-c ratio was 3.64 *versus* 0.33 in 16-week high fat compared to normal diet; p<0.001), while there were only trends (but not significant) for higher plasma levels of carbohydrates (increased by 41% compared to normal diet), including glucose (Table 2). We did not perform glucose tolerance testing, but the plasma levels of glucose only slightly increased over time, suggesting preserved glucose tolerance in these animals. Plasma levels of creatine phosphokinase were increased (Table 2).

**Table 2:** Body weight and biochemical parameters before and after high fat and normal diet feeding during 16 weeks in *Psammomys obesus* gerbils.

	BASELINE			AFTER 16 WEEKS		
	Normal diet (n = 10)	High fat diet (n = 10)	p-values	Normal diet (n = 10)	High fat diet (n = 10)	p-values
**Body weight** (in g)	93 ± 6	96 ± 2	**NS** (p = 0.537)	99 ± 8	123 ± 3	** (p = 0.002)
**Glucose** (in g/L)	0.62 ± 0.06	0.60 ± 0.04	**NS** (p = 0.648)	0.55 ± 0.08	0.78 ± 0.10	**NS** (p = 0.153)
**Triglycerides** (in g/L)	0.51 ± 0.06	0.80 ± 0.08	**NS** (p = 0,250)	0.60 ± 0.06	1.04 ± 0.31	* (p = 0.035)
**Total cholesterol** (in g/L)	0.58 ± 0.09	0.60 ± 0.04	**NS** (p = 0,750)	0.82 ± 0.07	5.50 ± 1.49	* (p = 0.043)
**LDL cholesterol** (in g/L)	0.12 ± 0.03	0.12 ± 0.01	**NS** (p = 0.953)	0.15 ± 0.05	4.19 ± 1.38	* (p = 0.033)
**HDL cholesterol** (in g/L)	0.38 ± 0.07	0.37 ± 0.02	**NS** (p = 0.865)	0.46 ± 0.08	1.15 ± 0.14	** (p = 0.003)
**CPK** (in IU/L)	317 ± 25	424 ± 63	**NS** (p = 0.206)	451 ± 64	759 ± 177	* (p = 0.025)

Values are given as means ± SEM.

** 0.001<P<0.05

*0.01<p<0.05 high fat diet *versus* normal diet at baseline and after 16 –week feeding.

*Abbreviations*. LDL, low-density lipoprotein; HDL, high-density lipoprotein; CPK, creatine phosphokinase; NS, not significant.

### Myocardial architecture and morphometry

**Fig 1:**
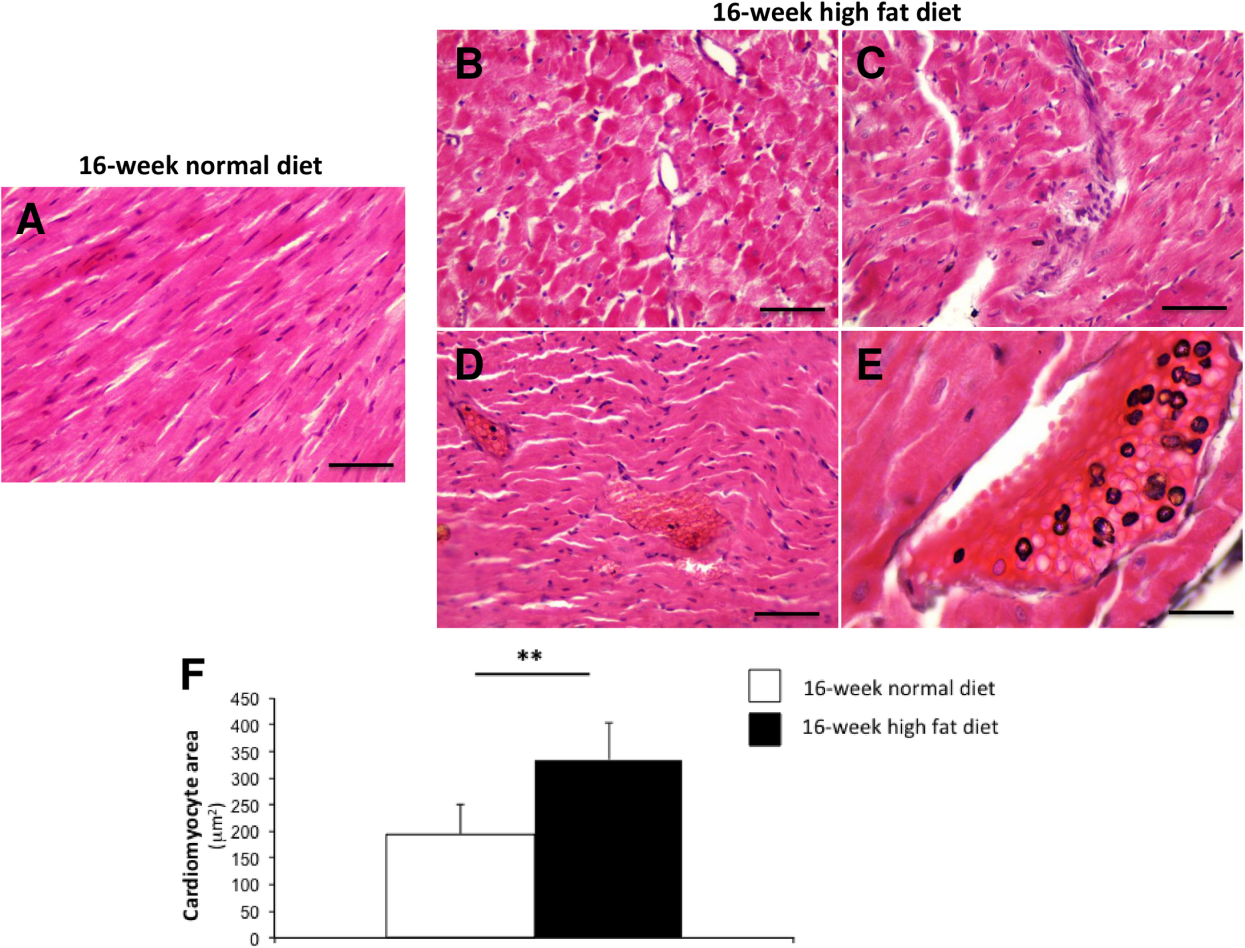
Representative haematoxylin and eosin-stained myocardial sections from *Psammomys obesus* gerbils fed with normal diet (A) or high fat dieting (B, C, D, E) during 16 weeks. Myocardial sections were obtained at 400-fold (A, B, C, D; Scale bars: 50μm) and 1000-fold (E; Scale bar: 20μm) magnification. Cardiomyocyte hypertrophy (assessed by cardiomyocyte area in square micrometer) was exacerbated in the heart from *Psammomys obesus* gerbils fed with high fat diet (n = 10; black bars) compared to *Psammomys obesus* gerbils fed with normal diet (n = 10; white bars) during 16 weeks (F). Values are expressed as mean ± SEM. **0.001<P<0.01 high fat *versus* normal diet fed *Psammomys obesus* gerbils.

In 16-week normal diet fed animals, myocardial architecture was normal, with myofibrils and muscle bundles (Fig 1A). High fat diet induced structural disorganization and interstitial edema (Fig 1B and 1C), associated with the accumulation of infiltrating cells (Fig 1C) and lipids (Fig 1D and 1E) within the myocardium, suggesting cardiomyopathy. This was associated with cardiomyocyte hypertrophy characterized by the increase in cell surface area (Fig 1B, 1C and 1F).

**Fig 2:**
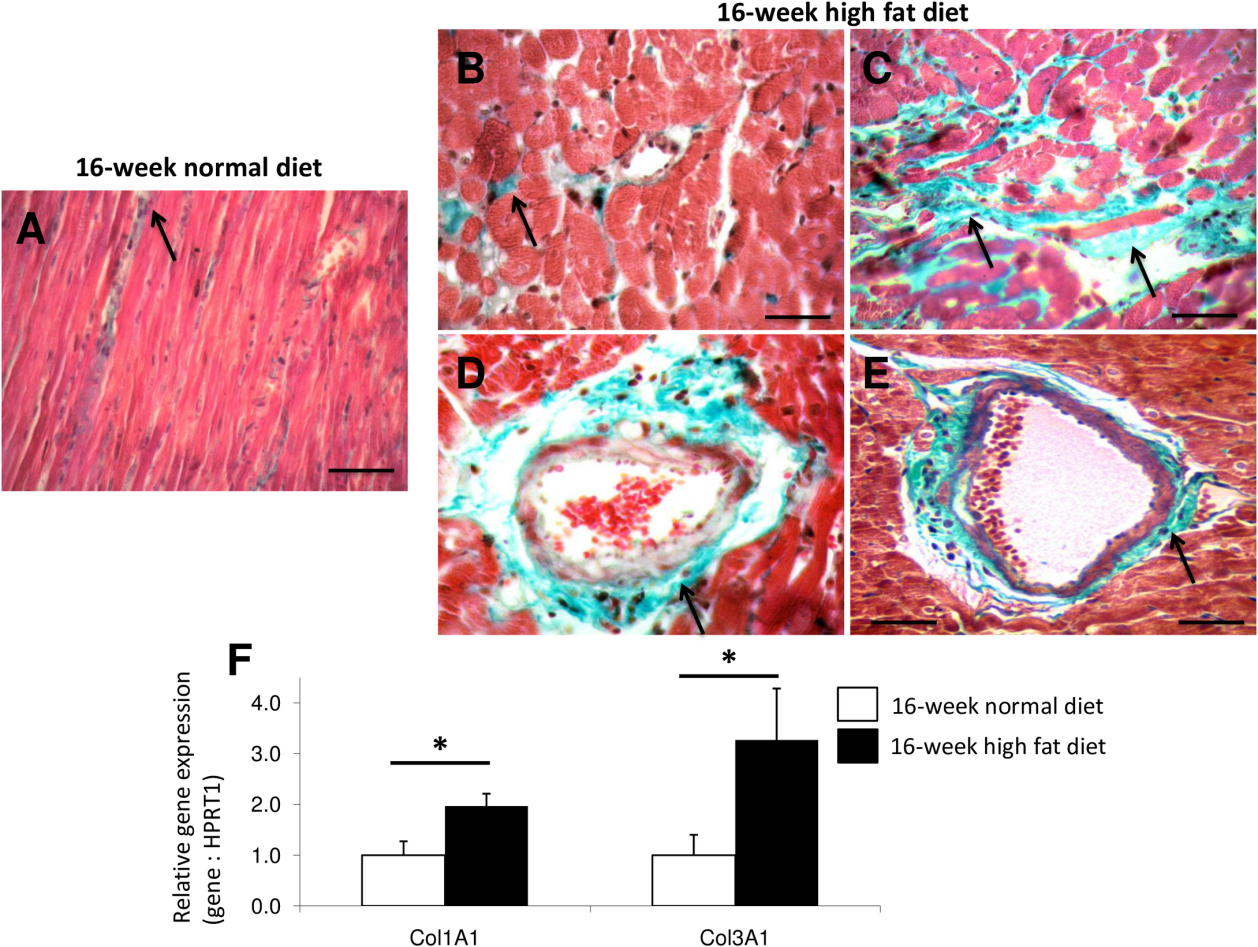
Representative Masson Trichrome stained myocardial sections from *Psammomys obesus* gerbils fed with normal diet (A) or high fat diet (B, C, D, E) during 16 weeks. Trichrome Masson staining was performed to detect fibrotic areas (in green; indicated by arrows). Sections were obtained at 400-fold. Scale bars: 50μm. Relative myocardial gene expressions of pro-alpha 1 chains of collagen type I (Col1A1) and 3 (Col3A1) in 16-week normal (n = 10; white bars) and high fat (n = 10; black bars) diet fed *Psammomys obesus* gerbils (F). Values are expressed as mean ± SEM. *0.01<P<0.001 high fat *versus* normal diet fed *Psammomys obesus* gerbils.

Because fibrosis and collagen deposition influence the mechanical properties of the myocardium, we used Masson’s Trichrome staining to detect fibrotic area. High fat diet exacerbated myocardial interstitial and perivascular fibrosis (Fig 2A–2E). This was associated with increased myocardial gene expressions of pro-α1 chains of collagen type 1 (Col1A1) and 3 (Col3A1) (Fig 2F).

### Myocardial infiltration by neutrophils [myeloperoxidase (MPO)-positive cells]

**Fig 3:**
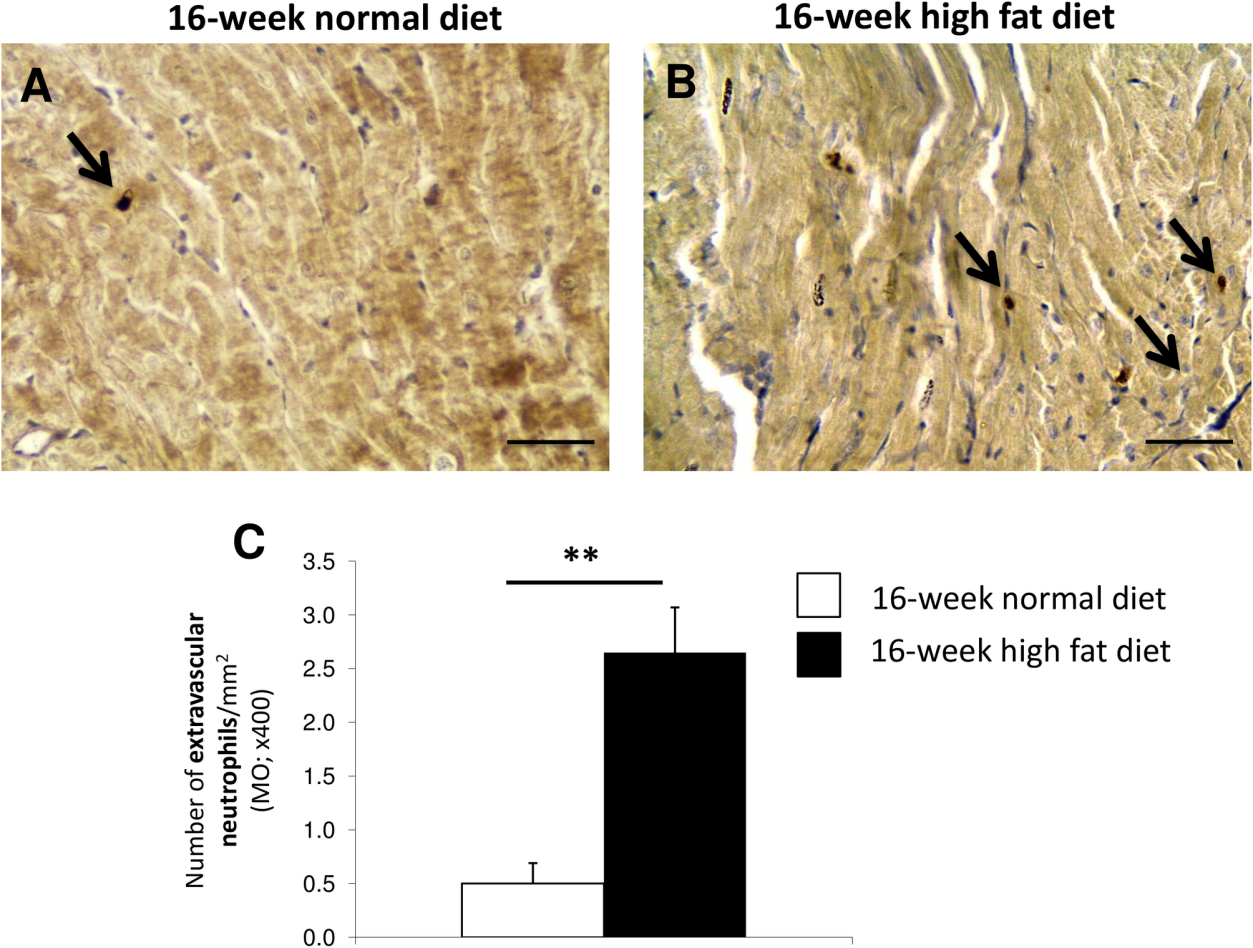
Characterization of inflammatory cells infiltrating the myocardium in 16-week high fat diet-fed *Psammomys obesus* gerbils. Neutrophil [myeloperoxidase (MPO)-positive stained cells] immunostaining in myocardial sections from *Psammomys obesus* gerbils fed with normal (**A**) or high fat diet (**B**) during 16 weeks. Arrows indicate MPO-positive cells. Scale bars: 50 μm. The number of extravascular MPO-positive cells was determined in myocardial sections from 16-week normal diet (n = 10; white bars) and high fat (n = 10; black bars) diet fed *Psammomys obesus* gerbils (**C**). Values are expressed as mean ± SEM. **0.001<P<0.01 high fat *versus* normal diet fed *Psammomys obesus* gerbils.

To characterize the inflammatory cells infiltrating the myocardium, we performed immunohistochemistry for myeloperoxidase (MPO) to detect the presence of neutrophils. As illustrated in Fig 3, high fat diet increased the number of extravascular neutrophils per mm^2^ in the myocardium.

### Myocardial activation of inflammatory and apoptosis processes

**Fig 4:**
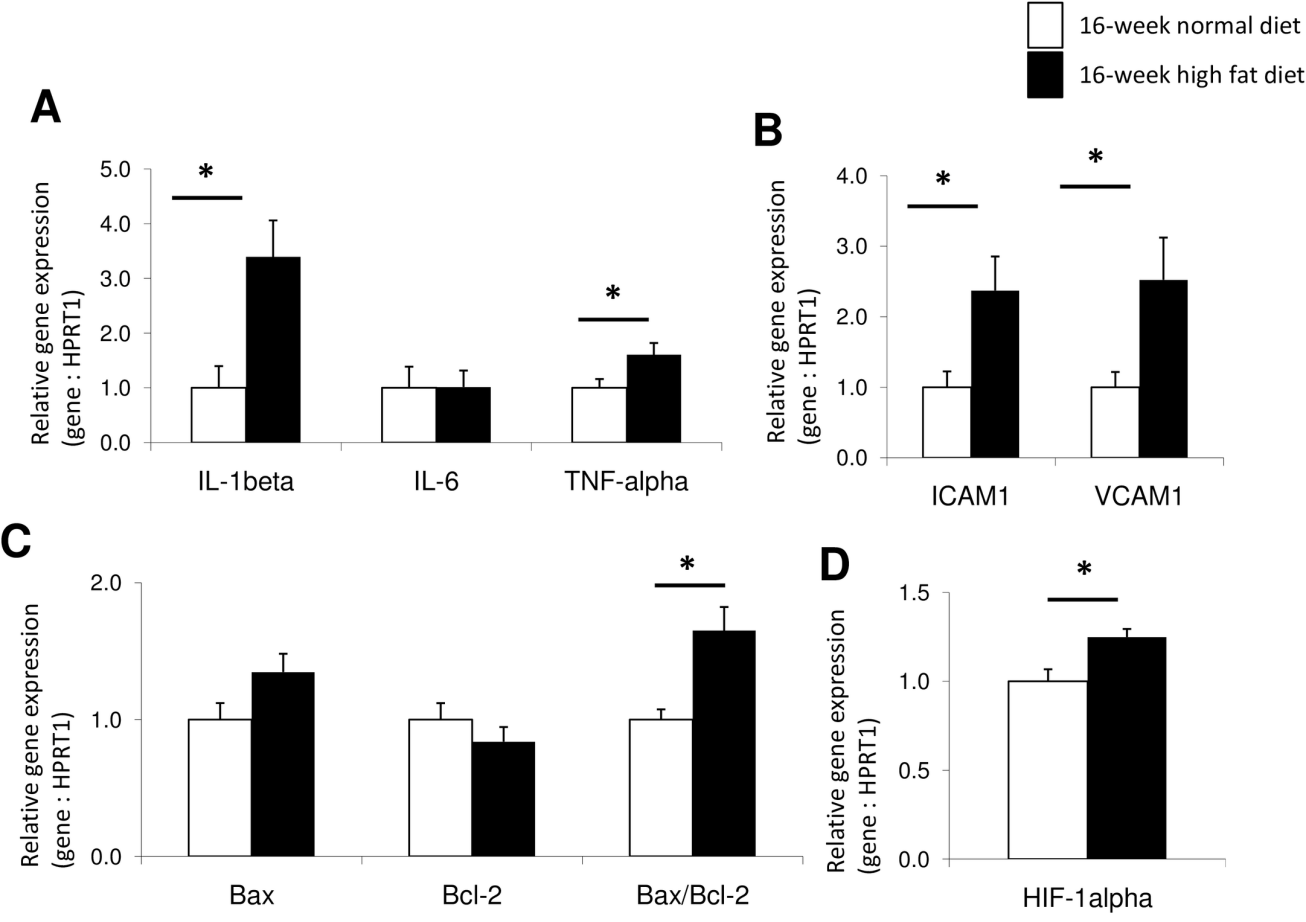
Myocardial relative expression of genes implicated in the activation of inflammatory processes (A, B), including the interleukin-1beta (IL-1β), the interleukin-6 (IL-6), the tumor necrosis factor-alpha (TNF-α), the intercellular adhesion molecule 1 (ICAM1) and the vascular cell adhesion molecule 1 (VCAM1); of apoptotic pathways (C), including the pro-apoptotic Bax and the anti-apoptotic Bcl-2 mitochondrial members and the resulting pro-apoptotic Bax/Bcl-2 ratio; and in the tissue response to hypoxia exposure (D), including the hypoxia-inducible factor-alpha (HIF-1α) in 16-week normal (n = 10; white bars) *versus* high fat (n = 10; black columns) diet fed *Psammomys obesus* gerbils. Values are expressed as mean ± SEM. * 0.01<P<0.05, high fat diet- *versus* normal diet-fed *Psammomys obesus* gerbils.

To assess if these increased numbers of inflammatory cells infiltrating the myocardium were associated with increased expressions of pro-inflammatory cytokines, we performed RTQ-PCR on myocardial samples. After 16-week high fat diet, there were increased myocardial gene expressions of IL-1β and TNF-α, while myocardial expression of IL-6 remained unchanged (Fig 4A). This was associated with enhanced expressions of cell adhesion molecules, including ICAM1 and VCAM1 in the myocardium (Fig 4B).

High fat diet increased the pro-apoptotic Bax-to-Bcl-2 gene ratio (Fig 4C), suggesting myocardial activation of apoptotic processes. To evaluate the presence of local hypoxic exposure of myocardial tissue in these animals, we assessed HIF-1α expression, which was increased after high fat diet (Fig 4D).

### Myocardial expression of molecules implicated in Ca^2+^ handling and contractile apparatus

**Fig 5:**
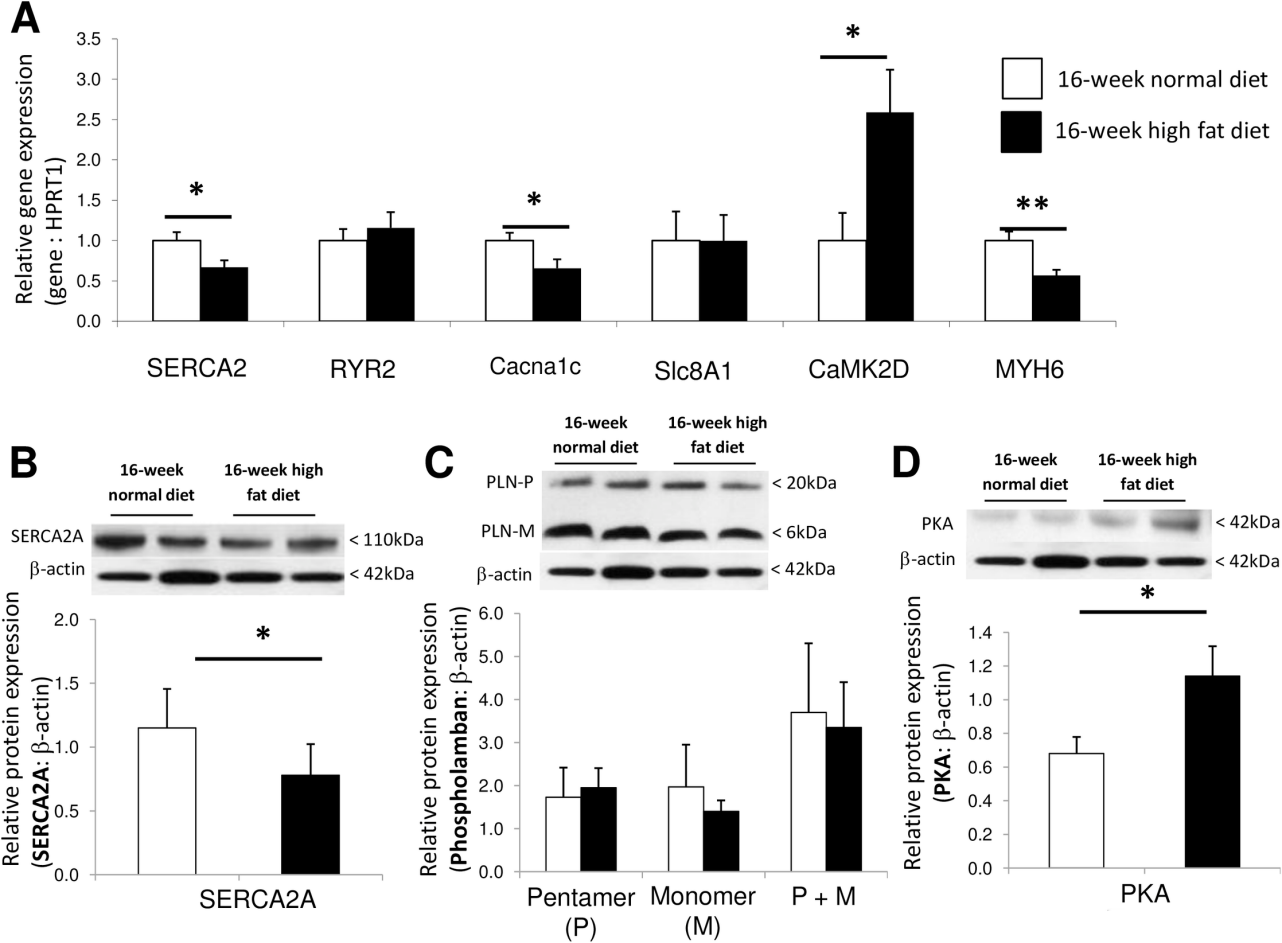
Myocardial relative expressions of genes implicated in calcium (Ca^2+^) handling implicated in cardiac contraction/relaxation, including the sarco/endoplasmic reticulum Ca^2+^ ATPase 2 (SERCA2), the type 2 ryanodine receptor (RYR2), the voltage-dependent L-type Ca^2+^ channel alpha1c subunit (Cacna1c), the solute carrier family 8 (Na^+^/Ca^2+^ exchanger) member 1 (Slc8A1), the Ca^2+^/calmodulin-dependent protein kinase 2 δ (CaMK2D) and the myosin heavy chain isoform 6 (MYH6 or α-MHC) in 16-week normal (n = 10; white bars) and high fat (n = 10; black bars) diet fed *Psammomys obesus* gerbils (A). Myocardial expressions of proteins regulating sarco/endoplamic calcium (Ca^2+^) handling implicated in cardiac contraction/relaxation cycle, including the sarco/endoplasmic reticulum Ca^2+^-ATPase 2 (SERCA2; B), phospholamban (PLN) pentamer (P) and monomer (M) (C) and PKA catalytic subunits (C-α; C) protein contents in 16-week high fat (n = 10; black bars) *versus* normal (n = 10; white bars) diet fed animals. Values are expressed as mean ± SEM. * 0.01<P<0.05; ** 0.001<P<0.01 high fat *versus* normal diet fed *Psammomys obesus* gerbils.

As illustrated in Fig 5A and 5B, expressions of SERCA2 (at gene and protein levels) and Cacna1c (at gene level) decreased in the hearts of 16-week high fat diet fed animals, suggesting altered myocardial Ca^2+^ handling. Myocardial gene expression of a cardiac muscle structure protein, the MYH6 (also called α-MHC) decreased after 16-week high fat diet (Fig 5A), while RYR2 and Slc8A1 expressions remained unchanged (Fig 5A), as well as protein levels of PLN pentamer and monomer (Fig 5C). The PLN exists in equilibrium between its monomeric and its pentameric forms. The PLN monomer is able to inhibit SERCA2 function, while the PLN pentamer acts as an inactive reservoir that is unable to interact with SERCA2A [25]. In addition, myocardial expressions of CaMK2D (Fig 5A) and PKA (Fig 5D) were higher, respectively at gene and protein levels.

## Discussion

The present results show that a relatively short period of high fat diet in *Psammomys obesus* gerbils, wild rodents with a genetic predisposition to develop obesity, diabetes and metabolic syndrome, results in severe alterations of cardiac structure, myocardial activation of inflammatory and apoptotic processes, and altered expressions of Ca^2+^-cycling proteins, including decreased expressions of SERCA2 and Cacna1c, increased expression CaMK2D, but no changes in RYR, phospholamban and Slc8a1 expressions.

*Psammomys obesus* gerbils quickly develop obesity and type 2 diabetes after standard laboratory chow diet during at least 12 weeks [18, 19]. In the present study, 16-week feeding with normal plant diet supplemented with high fat resulted in the development of obesity, associated with increased plasma lipid levels, but no significant changes (but a 42% increased trend) in plasma level of glucose. Here, we did not perform glucose tolerance testing, but the plasma levels of glucose only slightly increased over time, suggesting preserved glucose tolerance in these animals. These apparent discrepancies in diabetes development have already been described in *Psammomys obesus* gerbils removed from their natural halophilic plant diet [26]. Consistently with previous studies [27, 28], 16-week high fat diet increased plasma levels of triglycerides and cholesterol, together with increased levels of LDL and HDL cholesterol. The LDL-to-HDL cholesterol ratio was 4-fold increased with high fat diet, which strongly suggests an increased risk of cardiovascular diseases in these animals [29, 30].

Increased levels of circulating free fatty acids lead to their increased uptake and storage in non-adipose tissues, such as the heart [31]. Indeed, we found in high fat diet fed animals myocardial lipid accumulation, which is a source of fatty acids in excess of the cellular oxidative needs. Here, we showed upregulated plasma CPK levels after 16-week high fat diet, suggesting muscle damage. This may result from cardiac muscle injury caused by oxidative stress induced by diet-induced hyperlipidemia state, as previously shown [32]. Fatty acyl-coA derived from triglycerides may be shunted into non-oxidative metabolic pathways, resulting in the accumulation of toxic lipid intermediates, which may contribute to lipotoxicity, including contractile abnormalities and apoptosis [33, 34].

After high fat diet, histological examination of the heart revealed signs of hypertrophy, inflammation, and fibrosis in interstitial and perivascular areas. Liu et al. previously reported that 8-week cholesterol-enriched diet markedly increased leucocyte MPO content and enhanced leucocyte MPO activity in rabbits [35]. Recruited inflammatory cells have been shown to act on fibroblasts, which play a central role in the progression of fibrosis by their ability to produce pro-collagen [36, 37]. This is consistent with our data showing fibrosis together with increased number of inflammatory cells (MPO-positive) infiltrating the myocardium and upregulated pro-collagen expression in the heart after high fat feeding. We also found increased myocardial expressions of pro-inflammatory cytokines (such as IL-1β and TNF-α) and adhesion molecules (such as ICAM1 and VCAM1). TNF-α is involved in many inflammatory responses, which can induce the release of many cytokines and functions as a chemotactic molecule to recruit neutrophils and monocytes. TNF-α also induces the expression of adhesion molecules by vascular endothelial cells. Moreover, LDL cholesterol contributes to the increased expressions of ICAM1 and VCAM1 on vascular cells [38, 39]. In the heart, TNF-α can also cause Ca^2+^ overload and affect therefore systolic and diastolic function [40]. TNF-α also induces overexpression of inducible nitric oxide (NO) synthase, leading to NO overproduction and contributing to the inhibition of normal myocardial contraction [41].

Myocardial accumulation of fatty acid metabolites and activation of inflammatory processes are also known to promote apoptosis [42]. Hypercholesterolemia has been associated with reduced myocardial anti-apoptotic Bcl-2 expression, which is known to have antioxidant properties [43]. Our results showed increased pro-apoptotic Bax-to-Bcl-2 ratio in the heart of the animals fed with high fat diet, suggesting activation of apoptotic processes. Myocardial apoptosis has been shown to contribute to cardiac insufficiency in many cardiovascular diseases, including cardiomyopathy. Acting upstream of the mitochondria, Bcl-2 family regulates the permeability of the mitochondrial membrane, thus the activation of downstream caspases and of apoptosis, through a tight regulation of the balance between pro-apoptotic Bax and anti-apoptotic Bcl-2 members [44]. This was associated with increased myocardial expression in HIF-1α in the animals fed with high fat diet, indicating local myocardial ischemia.

High fat feeding is associated with myocardial hypertrophy and fibrosis, but may eventually result in altered contractility. Although the phenomenon may have already been known to Virchow in 1973 [43], when he described “fatty metamorphosis” of the heart, the concept of cardiac “lipotoxicity” re-emerged only recently with its description in the heart of the obese Zucker diabetic fatty rat [13]. However, the mechanisms responsible for impaired contractile function of the heart induced by high fat diet remain obscure. In cardiomyocytes, intracellular Ca^2+^ level is tightly regulated by a balanced release and removal of Ca^2+^ into the cytosol. Cytosolic Ca^2+^ is sequestered into the sarcoplasmic lumen by the SERCA2, whose activity is reversibly regulated by PLN [44]. Phosphorylation of PLN (by PKA or CAMK2D) relieves its inhibitory effects on SERCA2 and thus modulates cardiac contractility, but also survival and remodeling [45]. Here, we showed that high fat diet decreased myocardial expressions of SERCA2 and Cacna1, while PLN, RYR2 and Slc8A1 expressions did not change. This suggests altered sarcoplasmic Ca^2+^ cycling, which could account for cardiac contractile dysfunction. Decreased SERCA2 content responsible for decreased sarcoplasmic Ca^2+^ uptake during relaxation has been shown to result in impaired myocardial relaxation and diastolic dysfunction, finally leading to overt heart failure [46]. Downregulation of SERCA2 gene expression by pro-inflammatory cytokines, such as TNF-α[47, 48] may indeed cause cardiac diastolic dysfunction by decreasing diastolic Ca^2+^ reuptake [44, 47, 49].

In the present study, we also found overexpression of PKA and CaMK2D after high fat feeding. These molecules have been implicated in cardiac excitation–contraction coupling, at least partly through their effects on the inactivation of PLN. Here, we did not evaluate the phosphorylated levels of PLN. However, heart failure has been associated with increased CaMK2D expression, implicated in decreased cardiac contractile properties, uncoupled cardiac excitation-contraction and possible sudden death [50]. Transgenic mice overexpressing myocardial CaMK2D showed a marked hypertrophy, altered expressions and phosphorylation levels of RYR2 and PLN [50]. This strongly suggested that decreased expressions of PKA and CaMK2D should contribute to the alteration of myocardial function after high fat feeding.

Here, we found decreased myocardial expression of MYH6 (or α-MHC) after high fat feeding. This is consistent with previous data showing that downregulation of SERCA2 activity is accompanied by a switch from the α to the β isoform of MHC that would be expected to impair contractile mechanics [51, 52]. These contractile protein isoforms have different efficiencies, with the α-isoform presenting the fast and less efficient isoform, whereas MHC-β is the slow and more efficient isoform [53]. Upregulated β-MHC and decreased SERCA2 levels have been associated with diastolic dysfunction, but preserved systolic function in diabetic cardiomyopathy [45], suggesting that cardiac contractile dysfunction results from the combined effects of altered Ca^2+^ handling and altered MHC expression. However, even if altered expression of biological determinants seem to be in favor of major myocardial anomalies of Ca^2+^ transients and subsequent heart dysfunction [54], we did not evaluate the dynamics of the Ca^2+^ transients and the cardiac function.

Although high fat diet-induced cardiovascular effects have been described differently in males and females [55, 56], we were unable to compare sex-related myocardial effects in the present study, because of the small number of male gerbils we get. This needs to be further evaluated.

We conclude that a few weeks high fat diet in desert rats may be a cause of cardiomyopathy characterized by inflammation, apoptosis, ischemia and altered calcium handling. Further investigations are necessary to determine if these structural and pathobiological alterations are implicated in cardiac dysfunction in this experimental model.

## Supporting Information

S1 File Raw data—Sahraoui_Biochemical parameters. (XLS)

S2 File Raw data—Sahraoui_RTQPCR_Biology. (XLS)
